# Appraising the causal relationship between plasma caffeine levels and neuropsychiatric disorders through Mendelian randomization

**DOI:** 10.1186/s12916-023-03008-0

**Published:** 2023-08-08

**Authors:** Benjamin Woolf, Héléne T. Cronjé, Loukas Zagkos, Stephen Burgess, Dipender Gill, Susanna C. Larsson

**Affiliations:** 1 School of Psychological Science, University of Bristol, Bristol, UK; 2 MRC Integrative Epidemiology Unit, University of Bristol, Bristol, UK; 3 MRC Biostatistics Unit at the University of Cambridge, Cambridge, UK; 4 Department of Public Health, Section of Epidemiology, University of Copenhagen, Copenhagen, Denmark; 5 Department of Epidemiology and Biostatistics, School of Public Health, Imperial College London, London, UK; 6 Unit of Medical Epidemiology, Department of Surgical Sciences, Uppsala University, Uppsala, Sweden; 7 Unit of Cardiovascular and Nutritional Epidemiology, Institute of Environmental Medicine, Karolinska Institutet, Stockholm, Sweden

**Keywords:** Anorexia nervosa, Bipolar disorder caffeine, Coffee, Depression, Mendelian randomization, Schizophrenia

## Abstract

**Background:**

Caffeine exposure modifies the turnover of monoamine neurotransmitters, which play a role in several neuropsychiatric disorders. We conducted a Mendelian randomization study to investigate whether higher plasma caffeine levels are causally associated with the risk of anorexia nervosa, bipolar disorder, major depressive disorder (MDD), and schizophrenia.

**Methods:**

Summary-level data on the neuropsychiatric disorders were obtained from large-scale genome-wide association studies (GWASs) of European ancestry participants (*n* = 72,517 to 807,553) and meta-analyzed with the corresponding data from the FinnGen study (*n* = 356,077). Summary-level data on plasma caffeine were extracted from a GWAS meta-analysis of 9876 European ancestry individuals. The Mendelian randomization analyses estimated the Wald ratio for each genetic variant and meta-analyzed the variant-specific estimates using multiplicative random effects meta-analysis.

**Results:**

After correcting for multiple testing, genetically predicted higher plasma caffeine levels were associated with higher odds of anorexia nervosa (odds ratio [OR] = 1.124; 95% confidence interval [CI] = 1.024–1.238, *p*_FDR_ = 0.039) and a lower odds of bipolar disorder (OR = 0.905, 95% CI = 0.827–0.929, *p*_FDR_ = 0.041) and MDD (OR = 0.965, 95% CI = 0.937–0.995, *p*_FDR_ = 0.039). Instrumented plasma caffeine levels were not associated with schizophrenia (OR = 0.986, 95% CI = 0.929–1.047, *p*_FDR_ = 0.646).

**Conclusions:**

These Mendelian randomization findings indicate that long-term higher plasma caffeine levels may lower the risk of bipolar disorder and MDD but increase the risk of anorexia nervosa. These results warrant further research to explore whether caffeine consumption, supplementation, or abstinence could render clinically relevant therapeutic or preventative psychiatric effects.

**Supplementary Information:**

The online version contains supplementary material available at 10.1186/s12916-023-03008-0.

## Background

Caffeine is a widely consumed central nervous system stimulant found in coffee, tea, and cacao and is added to certain soft drinks, energy drinks, and analgesic drugs. The effects of caffeine are mainly mediated through the blockade of adenosine A_1_ and A_2_ receptors in the brain, which results in increased turnover of monoamine neurotransmitters, such as serotonin, dopamine, and noradrenaline [1]. Dysregulation of the monoamine neurotransmitter systems plays a role in several neuropsychiatric disorders, including anorexia nervosa [2], bipolar disorder [3], depression [4], and schizophrenia [5]. Furthermore, caffeine consumption is lower in people with depressive symptoms than in non-depressed individuals [6, 7], whereas caffeine consumption is higher in those with schizophrenia than in the general population [8]. However, whether caffeine consumption is causally associated with the risk of developing neuropsychiatric disorders, or whether these disorders affect caffeine consumption, remains unestablished. Moreover, tolerance develops to some but not to all effects of caffeine [1]. Thus, whether long-term higher circulating caffeine levels affect the risk of neuropsychiatric disorders remains unclear.

The metabolism of caffeine is highly variable among individuals and depends in part on genetic variations in the activity of the enzyme cytochrome P450 1A2 (CYP1A2), which is responsible for over 90% of caffeine metabolism. For example, genetic variants in the *CYP1A2* gene are associated with a lower paraxanthine to caffeine ratio (which reflects slower caffeine metabolism), higher plasma caffeine levels, and lower habitual caffeine consumption [9]. The latter association is likely related to the fact that persons with a genetic predisposition to slower caffeine metabolism require lower amounts of caffeine to reach the desired psychostimulant effect compared to individuals with faster caffeine metabolism.

To investigate whether long-term higher caffeine exposure causally associates with the risk of neuropsychiatric disorders, we conducted a Mendelian randomization (MR) study of the association between genetically predicted plasma caffeine levels and the risk of anorexia nervosa, bipolar disorder, major depressive disorder (MDD), and schizophrenia. Using genetic variants to instrument an exposure of interest (e.g., plasma caffeine) through MR reduces the biases that often limit observational studies, including reverse causation, as genetic variants cannot be modified by the outcome (such as neuropsychiatric disorders).

## Methods

### Study overview

**Fig. 1:**
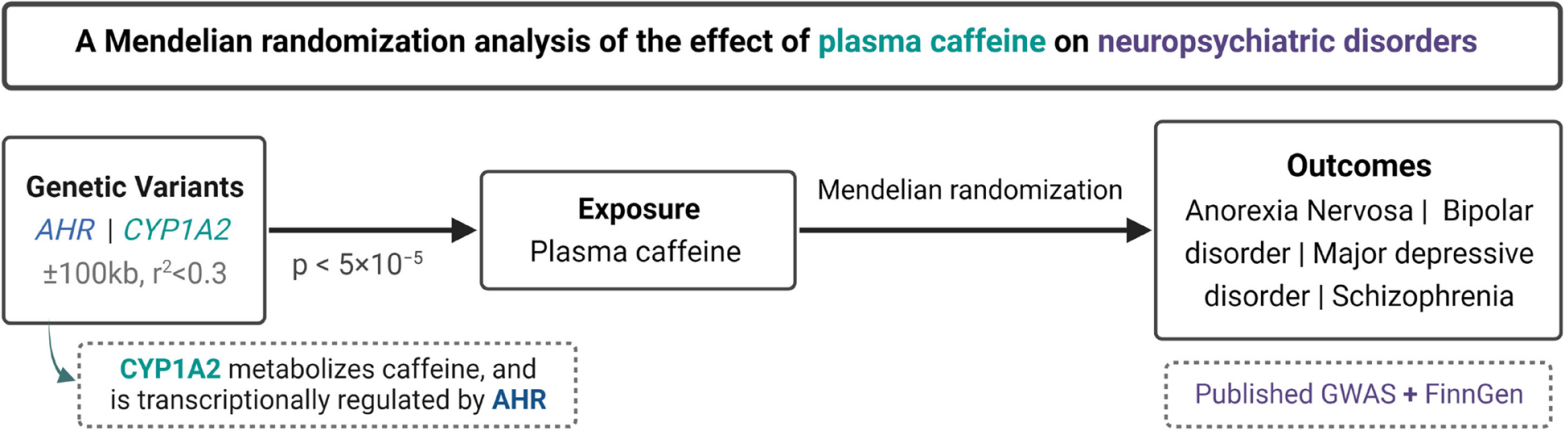
Study design overview (created with BioRender.com). AHR, aryl hydrocarbon receptor; CYP1A2, cytochrome P450 1A2 (CYP1A2); GWAS, genome-wide association study

This study applied a two-sample MR design with publicly available summary-level data taken from meta-analyses of genome-wide association studies (GWASs) as well as the FinnGen study, which was not included in any of the GWAS meta-analyses (Fig. 1). Outcomes included in this study were anorexia nervosa, bipolar disorder, MDD, and schizophrenia. Brief information about the used data sources for plasma caffeine and outcomes are described below. More comprehensive details for each GWAS are described in the corresponding articles [9–14].

### Data sources

#### Plasma caffeine GWAS

Cornelis et al. performed a meta-analysis of summary statistics from six plasma caffeine (1,3,7-trimethylxanthine) GWASs comprising 9876 participants of mainly European ancestry, with mean ages between 47 and 71 years [9, 15]. Participants were asked to fast prior to measurements being taken, and each study standardized plasma caffeine measurements. Association estimates accounted for confounding by smoking status and, when applicable, age, sex, and principal components of ancestry.

#### Anorexia nervosa GWAS

Watson et al.’s GWAS was a meta-analysis from the Psychiatric Genetic Consortium (PGC) Eating Disorders Working Group, which contained 16,992 cases of anorexia nervosa and 55,525 controls from 33 studies—mostly of European ancestry individuals [14, 16]. Studies were asked to adjust their analyses for the first five principal components of ancestry. The linkage disequilibrium score regression intercept for the GWAS implied the presence of an insignificant residual population structure.

#### Bipolar disorder GWAS

This study is a meta-analysis of 41,917 cases of bipolar disorder and 371,549 controls from the PGC and five population-based cohort studies including the UK Biobank (UKB) [16, 17]. Samples were restricted to participants of European ancestry, and cases were diagnosed using the Diagnostic and Statistics Manual or International Classification of Diseases guidelines. The genomic control inflation factor was between 0.97 and 1.05 for the participating studies, implying minimal residual population structure.

#### MDD GWAS

Howard et al. meta-analyzed GWAS summary statistics of 170,756 MDD cases and 329,443 controls from the PGC and the UK Biobank [11]. All study samples largely comprised individuals of European ancestry. Cases were identified using a combination of self-report and medical records. The linkage disequilibrium (LD) score regression intercept for the GWAS summary statistics used is 1.02 (standard error = 0.01) implying the presence of insignificant residual population structure. GWAS summary statistics were extracted from the OpenGWAS platform using the ID: ieu-b-102 [18, 19].

#### Schizophrenia GWAS

Trubetskoy et al. meta-analyzed genetic data from 90 cohorts within the PGC [10, 16]. The resulting sample included 76,755 schizophrenia cases and 243,649 controls. Participating studies used genomic quality control to control for inflated test statistics and adjusted for at least four principal components of ancestry.

#### FinnGen study

The FinnGen study is a large (*n* = 356,077 in round 8) population biobank, based in Finland, described in detail elsewhere [20, 21]. GWAS summary data on the FinnGen cohort (round 8) includes 390 individuals with anorexia nervosa (FinnGen phenotype ID: R18 ANOREXIA), 6562 with bipolar disorder (F5 BIPO), 39,747 with MDD (F5 DEPRESSIO), and 6522 with schizophrenia (F5 SCHZPHR) [22]. Cases were identified from medical records [20, 21]. These GWASs adjusted for age, sex, the first 10 genetic principal components, and genotyping batch [12].

### Mendelian randomization analysis

We selected single-nucleotide polymorphisms (SNPs) that were strongly associated (*p* < 5 × 10^−5^) with plasma caffeine levels and located within 100 kb of the *CYP1A2* and aryl hydrocarbon receptor (*AHR*) gene regions as instrumental variables (GRCh37/hg19 assembly by Ensembl: 15:75041185-75048543 and 7:17338246-17385776, respectively). These genes were selected owing to their role in caffeine metabolism [9]. Variants in these gene regions have been used as instrumental variables for plasma caffeine in previous MR studies [23, 24]. The statistical significance threshold of *p* < 5 × 10^−5^ was selected to ensure that the used SNPs would be strong instruments for plasma caffeine, while accounting for gene region-wide (a Bonferroni correction for the 955 SNPs measured by Cornelis et al. within the two gene regions provides a *p* ~  < 5 × 10^−5^). SNPs were clumped with a *r*^2^ of 0.3 and 10,000 kb windows using the TwoSampleMR R package [25, 26]. We used the false discovery rate inverse quantile transformation (FIQT) Winner’s curse correction to account for Winner’s curse [27]. We harmonized data sources using TwoSampleMR and excluded palindromic SNPs, which could not be aligned based on their allele frequency.

We firstly meta-analyzed the SNP-outcome associations from each GWAS meta-analysis with the equivalent outcome data in the FinnGen study using an inverse variance weighted meta-analysis. Secondly, we conducted the MR analysis. The primary estimator was the Wald ratio, which was defined as the ratio of the SNP-outcome association to the SNP-exposure association. Wald ratios were combined using a multiplicative inverse variance-weighted (IVW) random effects model, using SNPs’ LD matrix estimated from the European subsample of the 1000 Genomes Project to account for correlations between variants [28]. This was implemented using the code for random effects IVW (accounting for correlations) by Burgess et al. [28]. A correlated variants IVW estimator was chosen over a more conventional (independent variant) IVW estimator because of the potential to improve the precision of MR estimates in a *cis* setting. We used the Benjamini–Hochberg procedure to correct for multiple testing. A false discovery rate (FDR) adjusted *p*-value < 0.05 was regarded as statistically significant. Odds ratios (OR) presented here represent the multiplicative increase in the odds for each standard deviation (SD) increase in plasma caffeine levels.

Heterogeneity tests have been proposed as sensitivity analyses for pleiotropy in MR studies [29]. Because these methods have not been extended to *cis* settings with correlated variants, we first explore the robustness of outlier SNPs using leave-one-out analyses and then explore heterogeneity between the AHR and CYP1A2 genes. We additionally explore the heterogeneity in the MR estimates between data sources and replicate our analysis using only the lead SNP from each gene region (rs4410790 for *AHR* and rs2472297 for *CYP1A2*). To the best of our knowledge, there is no sample overlap between the exposure and outcome GWASs.

## Results

After clumping and harmonization, we included 7 SNPs as instrumental variables for plasma caffeine in all analyses except for MDD, where 8 SNPs were included (Additional file 1: Tables S1-S3). The discrepancy in the number of SNPs is due to the different coverages of SNPs measured in the GWASs used. After accounting for LD among SNPs, these genetic instruments had an average *F* statistic of 44 and 25, respectively. This is consistent with approximately 2–4% bias in the MR estimates due to the use of weak instruments.

**Fig. 2:**
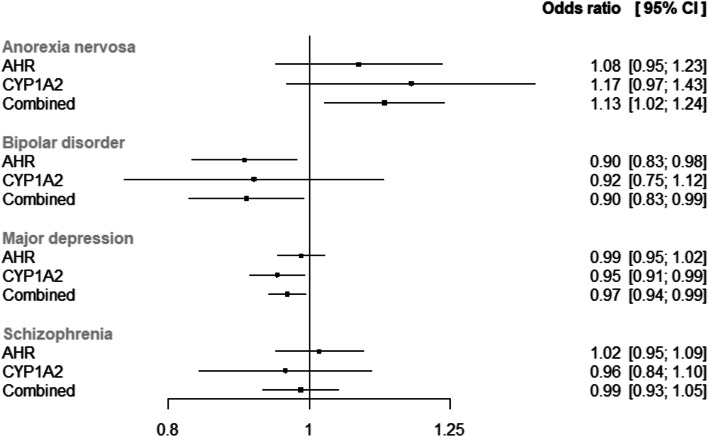
Forest plot of two-sample MR estimates. “OR” represents the effect of a standard deviation increase in genetically predicted plasma caffeine levels on the odds of each outcome. AHR, aryl hydrocarbon receptor; CI, confidence interval; CYP1A2, cytochrome P450 1A2 (CYP1A2); OR, odds ratio

Our MR analysis indicated that each SD increase in genetically predicted plasma caffeine was associated with a 1.124 (95% confidence interval [CI] = 1.024 to 1.238, *p*_FDR_ = 0.039) fold higher odds of anorexia nervosa (Fig. 2 and Additional file 1: Fig. S1). In contrast, there were inverse associations of genetically predicted plasma caffeine levels with bipolar disorder (OR = 0.905, 95% CI = 0.827 to 0.929, *p*_FDR_ = 0.041) and MDD (OR = 0.965, 95% CI = 0.937 to 0.995, *p*_FDR_ = 0.039). There was no evidence of an association with schizophrenia (OR = 0.986, 95% CI = 0.929 to 1.047, *p*_FDR_ = 0.646).

**Fig. 3:**
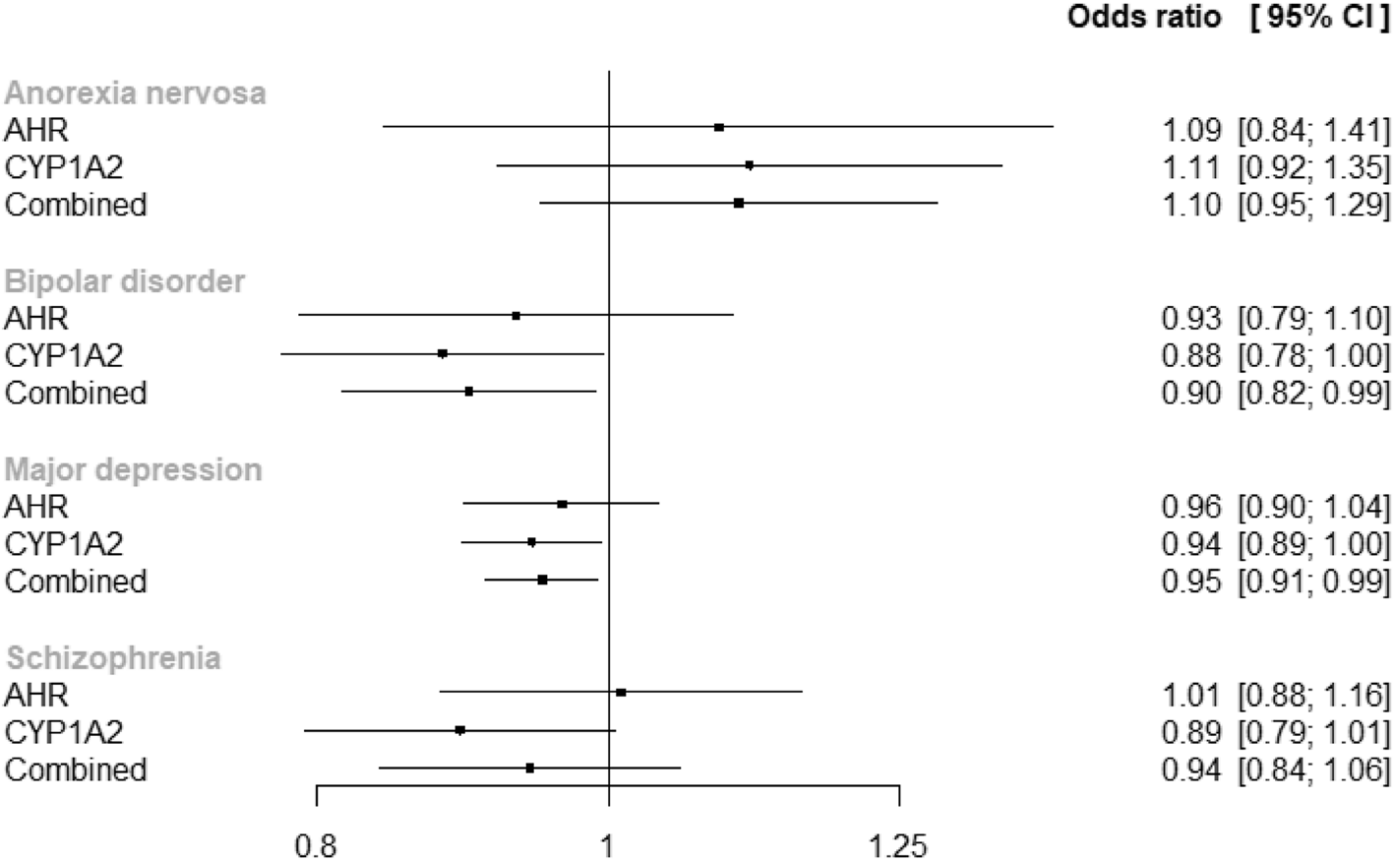
Forest plot of two-sample Mendelian randomization estimates using lead variants (rs4410790 for AHR and rs2472297 for CYP1A2) only. “OR” represents the effect of a standard deviation increase in genetically predicted plasma caffeine levels on the odds of each outcome. AHR, aryl hydrocarbon receptor; CI, confidence interval; CYP1A2, cytochrome P450 1A2 (CYP1A2); OR, odds ratio

Our leave-one-out analyses (Additional file 1: Fig. S2) found that exclusion of the variant rs12903896 resulted in deflation towards the null for the CYP1A2 anorexia estimate, but resulted in more extreme estimates, further away from the null, for the other outcomes. This effect was much less pronounced on the combined CYP1A2 and AHR estimates. All the AHR estimates were broadly consistent after the exclusion of each AHR SNP in the leave-one-out sensitivity analysis. The results of the analysis including only the lead variants for each gene (rs4410790 for AHR and rs2472297 for CYP1A2) are similar to those in our primary analysis (Fig. 3), other than being less precise. Together, these imply that rs12903896 has not biased our estimates.

**Fig. 4:**
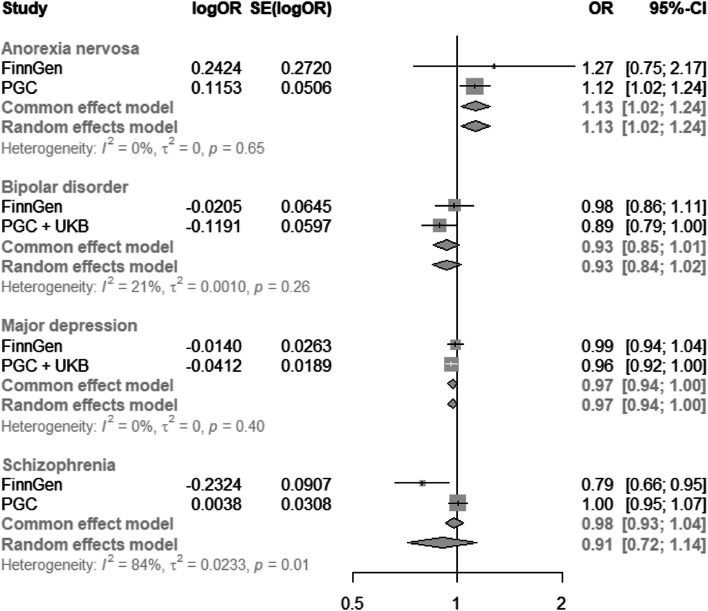
Heterogeneity in Mendelian randomization estimates from the two different data sources. “OR” represents the effect of a standard deviation increase in genetically predicted plasma caffeine levels on the odds of each outcome. PGC, Psychiatric Genetic Consortia; UKB, UK Biobank; CI, confidence interval; logOR, log odds ratio; OR, odds ratio

We did not find strong evidence of heterogeneity between AHR and CYP1A2 estimates for anorexia nervosa (*I*^2^ = 0%), bipolar disorder (*I*^2^ = 0%), MDD (*I*^2^ = 41%), or schizophrenia (*I*^2^ = 0%). We also did not find evidence of heterogeneity between the two data sources for anorexia nervosa, bipolar disorder, or MDD (Fig. 4). However, there was evidence of heterogeneity for schizophrenia (*I*^2^ = 84%, *p* = 0.01), although an additive random effects meta-analysis of the MR estimates from the PGC and FinnGen still did not find evidence of an effect (95% CI: 0.72 to 1.14).

## Discussion

This two-sample MR study found that genetically predicted long-term higher plasma caffeine levels were associated with a higher risk of anorexia nervosa, but a lower risk of bipolar disorder and MDD. We found no significant associations of genetically predicted plasma caffeine levels with the risk of schizophrenia.

The link between dietary caffeine intake and depressive symptoms has been reported in several observational, mainly cross-sectional studies, which were by nature of their design unable to investigate the direction of the association. A recent meta-analysis of observational studies reported that high versus low caffeine intake was associated with an overall 14% lower risk of future depressive symptoms in two cohort studies and an amalgamated 13% lower odds of depressive symptoms across 10 cross-sectional studies [7]. Our investigation confirms these findings and extends the evidence that higher plasma caffeine may play a causal role in reducing the risk of MDD as well as bipolar disorder for which epidemiological data on caffeine are scarce.

Caffeine may through adenosine antagonism play a role in anorexia nervosa as altered serotonergic and dopaminergic function is commonly observed in those suffering from this disorder [2]. Although anorexia nervosa patients often present with co-morbid psychiatric disorders such as MDD, the present MR study found that genetically predicted higher plasma caffeine was associated with an increased rather than decreased risk of anorexia nervosa. Since previous MR evidence is indicative of an association between higher plasma caffeine and lower body mass index [23], the observed effect may be a result of the more general association of plasma caffeine with weight loss. This novel finding warrants confirmation by further studies.

Although a positive association between caffeine intake and prevalent schizophrenia has been reported [8], we did not observe any association between genetically predicted plasma caffeine levels and schizophrenia risk. Thus, our finding suggests that caffeine is not a strong causal risk factor for this disorder and that findings in traditional epidemiological studies may arise spuriously due to residual confounding or reverse causation.

Summary data MR estimates should be interpreted as the average lifetime effect of greater exposure to caffeine [30]. However, the incidence of many mental disorders is not uniformly distributed across the lifespan. For example, anorexia nervosa is typically developed in adolescence or early adulthood. Future research may consider using longitudinal/individual-level data to explore whether this has implications for the presence of time-varying effects.

Our study has several strengths. The MR design reduces the risk of reverse causation bias and confounding by environmental factors. We further strengthen the validity of our findings by biologically plausible genetic instruments from genes known to affect caffeine metabolism and plasma caffeine levels. A priori gene selection additionally reduces the risk of confounding by pleiotropy. Finally, the use of large-scale GWAS data within a two-sample MR framework increased the statistical power of our analyses.

Nevertheless, despite the use of the largest publicly available GWAS data for the outcomes, we cannot exclude the possibility that we might have overlooked weak associations between plasma caffeine and the studied disorders. Furthermore, the possibility of genetic confounding by metabolites other than caffeine that are metabolized by the CYP1A2 enzyme cannot be ruled out. Although the use of a *cis* design should provide greater robustness through establishing the biological mechanism linking SNPs to the exposure [31], a limitation of it is that many standard MR sensitivity analyses cannot be used in settings with correlated variants. A further limitation is that the exposure GWAS adjusted for smoking status. Although adjustment for heritable covariates can create collider bias [32], the outcome associations were not adjusted for this variable. Therefore, while this adjustment may lead to bias, it will not affect the significance of MR estimates (as significance depends on the variant-outcome associations), and so inferences from our analyses should be valid [33, 34]. In addition, the use of summary-level data meant that dose–response relations of the effect of caffeine on psychiatric disorders could not be investigated. Finally, our MR analyses largely included data collected from individuals of European descent, and therefore, our findings may not be transferable to populations of non-European descent.

## Conclusions

This MR investigation provides evidence of possible causal relationships of long-term higher plasma caffeine levels with a higher risk of anorexia nervosa and a reduced risk of bipolar disorder and MDD. These results warrant further research to explore whether caffeine consumption, supplementation, or abstinence could render clinically relevant therapeutic or preventative psychiatric effects.

### Supplementary Information

**Additional file 1:** **Table S1.** Exposure related genome-wide association study summary data taken from Cornelis et al. **Table S2.** Summary-level data from FinnGen. **Table S3.** Genome-wide association study summary statistics taken from other data sources. **Table S4.** Meta-analyzed variant-outcome associations and Mendelian randomization Wald ratios. **Fig. S1.** X-Y scatter graphs of Mendelian randomization results. **Fig. S2.** Leave-one-out analyses.

## Data Availability

All data analyzed during the current study are available in Additional file 1: Tables S1 to S4. The R code is available at https://github.com/bar-woolf/applied-MR-code/blob/main/caff%20psyc.R. This study was not pre-registered.

## Abbreviations

AHR: Aryl hydrocarbon receptor
CI: Confidence interval
CYP1A2: Cytochrome P450 1A2
FDR: False discovery rate
GWAS: Genome-wide association study
IVW: Inverse variance-weighted
MDD: Major depressive disorder
MR: Mendelian randomization
OR: Odds ratio
PGC: Psychiatric Genetic Consortium
SNP: Single-nucleotide polymorphism
