# Supplementary material for: Appraising the causal relationship between plasma caffeine levels and neuropsychiatric disorders through Mendelian randomization
Source: BMC Med. 2023 Aug 8;21:296. doi: 10.1186/s12916-023-03008-0 (PMC10408049; doi:10.1186/s12916-023-03008-0)
Supplement: Supplementary file 1 — Additional file 1: Table S1. Exposure related genome-wide association study summary data taken from Cornelis et al. Table S2. Summary-level data from FinnGen. Table S3. Genome-wide association study summary statistics taken from other data sources. Table S4. Meta-analyzed variant-outcome associations and Mendelian randomization Wald ratios. Fig. S1. X-Y scatter graphs of Mendelian randomization results. Fig. S2. Leave-one-out analyses. [file 12916_2023_3008_MOESM1_ESM.docx]

Contents

[**Supplementary Tables** 2](#_Toc141292146)

[**Table S1. Exposure related genome-wide association study summary data taken from Cornelis *et al***. 3](#_Toc141292147)

[**Table S2. Summary-level data from FinnGen** 4](#_Toc141292148)

[**Table S3. Genome-wide association study summary statistics taken from other data sources** 6](#_Toc141292149)

[**Table S4: Meta-analyzed variant-outcome associations and Mendelian randomization Wald ratios** 8](#_Toc141292150)

[**Supplementary Figures** 10](#_Toc141292151)

[**Fig. S1. X-Y scatter graphs of Mendelian randomization results** 11](#_Toc141292152)

[**Fig. S2. Leave-one-out analyses** 15](#_Toc141292153)

# **Supplementary Tables**

## **Table S1. Exposure related genome-wide association study summary data taken from Cornelis *et al***.

| **Effect allele** | **Other allele** | **P-value** | **Chromosome** | **Position** | **Gene** | **SNP** | **Effect allele frequency** | **Beta** | **Standard error** |
| --- | --- | --- | --- | --- | --- | --- | --- | --- | --- |
| A | C | 1.075e-06 | 15 | 75017176 | CYP1A2 | rs2606345 | 0.337 | -0.073 | 0.015 |
| A | G | 1.862e-06 | 15 | 75018330 | CYP1A2 | rs35686934 | 0.046 | -0.162 | 0.034 |
| T | C | 1.003e-20 | 15 | 75027880 | CYP1A2 | rs2472297 | 0.215 | -0.161 | 0.017 |
| T | C | 6.646e-08 | 15 | 75052495 | CYP1A2 | rs12903896 | 0.595 | -0.078 | 0.014 |
| T | C | 1.814e-13 | 7 | 17284577 | AHR | rs4410790 | 0.385 | 0.107 | 0.015 |
| T | C | 4.811e-09 | 7 | 17303778 | AHR | rs10275488 | 0.118 | 0.129 | 0.022 |
| A | C | 2.123e-06 | 7 | 17399858 | AHR | rs10950657 | 0.450 | -0.068 | 0.014 |
| A | C | 1.139e-05 | 7 | 17464965 | AHR | rs73083829 | 0.851 | 0.088 | 0.020 |

## **Table S2. Summary-level data from FinnGen**

| **Chromosome** | **Position** | **Other allele** | **Effect allele** | **SNP** | **Nearest gene** | **P-value** | **Beta** | **Standard error** | **Alt allele frequency** | **Outcome** |
| --- | --- | --- | --- | --- | --- | --- | --- | --- | --- | --- |
| 7 | 17244953 | T | C | rs4410790 | AHR | 0.596 | -0.041 | 0.078 | 0.663 | R18_ANOREXIA |
| 7 | 17264154 | C | T | rs10275488 | AHR | 0.295 | 0.123 | 0.118 | 0.110 | R18_ANOREXIA |
| 7 | 17360234 | C | A | rs10950657 | RP11-507K12.1 | 0.134 | -0.113 | 0.076 | 0.605 | R18_ANOREXIA |
| 7 | 17425341 | C | A | rs73083829 | RP11-507K12.1 | 0.481 | 0.072 | 0.103 | 0.154 | R18_ANOREXIA |
| 15 | 74724835 | C | A | rs2606345 | CYP1A1 | 0.222 | 0.092 | 0.075 | 0.609 | R18_ANOREXIA |
| 15 | 74725989 | G | A | rs35686934 | CYP1A1 | 0.569 | -0.075 | 0.131 | 0.086 | R18_ANOREXIA |
| 15 | 74735539 | C | T | rs2472297 | CYP1A1 | 0.671 | 0.036 | 0.085 | 0.248 | R18_ANOREXIA |
| 15 | 74760154 | C | T | rs12903896 | CYP1A2 | 0.746 | -0.024 | 0.074 | 0.555 | R18_ANOREXIA |
| 7 | 17244953 | T | C | rs4410790 | AHR | 0.873 | -0.001 | 0.008 | 0.663 | F5_DEPRESSIO |
| 7 | 17264154 | C | T | rs10275488 | AHR | 0.965 | 0.001 | 0.013 | 0.110 | F5_DEPRESSIO |
| 7 | 17360234 | C | A | rs10950657 | RP11-507K12.1 | 0.895 | 0.001 | 0.008 | 0.604 | F5_DEPRESSIO |
| 7 | 17425341 | C | A | rs73083829 | RP11-507K12.1 | 0.645 | -0.005 | 0.011 | 0.154 | F5_DEPRESSIO |
| 15 | 74724835 | C | A | rs2606345 | CYP1A1 | 0.887 | -0.001 | 0.008 | 0.609 | F5_DEPRESSIO |
| 15 | 74725989 | G | A | rs35686934 | CYP1A1 | 0.467 | -0.010 | 0.014 | 0.086 | F5_DEPRESSIO |
| 15 | 74735539 | C | T | rs2472297 | CYP1A1 | 0.451 | 0.007 | 0.009 | 0.248 | F5_DEPRESSIO |
| 15 | 74760154 | C | T | rs12903896 | CYP1A2 | 0.989 | 0.000 | 0.008 | 0.555 | F5_DEPRESSIO |
| 7 | 17244953 | T | C | rs4410790 | AHR | 0.666 | 0.009 | 0.021 | 0.663 | F5_BIPO |
| 7 | 17264154 | C | T | rs10275488 | AHR | 0.906 | 0.004 | 0.031 | 0.110 | F5_BIPO |
| 7 | 17360234 | C | A | rs10950657 | RP11-507K12.1 | 0.479 | 0.014 | 0.020 | 0.604 | F5_BIPO |
| 7 | 17425341 | C | A | rs73083829 | RP11-507K12.1 | 0.843 | 0.005 | 0.027 | 0.155 | F5_BIPO |
| 15 | 74724835 | C | A | rs2606345 | CYP1A1 | 0.472 | -0.014 | 0.020 | 0.609 | F5_BIPO |
| 15 | 74725989 | G | A | rs35686934 | CYP1A1 | 0.809 | 0.008 | 0.035 | 0.086 | F5_BIPO |
| 15 | 74735539 | C | T | rs2472297 | CYP1A1 | 0.965 | 0.001 | 0.023 | 0.248 | F5_BIPO |
| 15 | 74760154 | C | T | rs12903896 | CYP1A2 | 0.806 | -0.005 | 0.020 | 0.555 | F5_BIPO |
| 7 | 17244953 | T | C | rs4410790 | AHR | 0.327 | 0.029 | 0.029 | 0.663 | F5_SCHZPHR |
| 7 | 17264154 | C | T | rs10275488 | AHR | 0.167 | -0.061 | 0.044 | 0.110 | F5_SCHZPHR |
| 7 | 17360234 | C | A | rs10950657 | RP11-507K12.1 | 0.855 | 0.005 | 0.028 | 0.604 | F5_SCHZPHR |
| 7 | 17425341 | C | A | rs73083829 | RP11-507K12.1 | 0.854 | -0.007 | 0.038 | 0.154 | F5_SCHZPHR |
| 15 | 74724835 | C | A | rs2606345 | CYP1A1 | 0.309 | 0.029 | 0.028 | 0.609 | F5_SCHZPHR |
| 15 | 74725989 | G | A | rs35686934 | CYP1A1 | 0.413 | 0.040 | 0.049 | 0.086 | F5_SCHZPHR |
| 15 | 74735539 | C | T | rs2472297 | CYP1A1 | 0.181 | 0.042 | 0.032 | 0.248 | F5_SCHZPHR |
| 15 | 74760154 | C | T | rs12903896 | CYP1A2 | 0.612 | 0.014 | 0.028 | 0.555 | F5_SCHZPHR |

## **Table S3. Genome-wide association study summary statistics taken from other data sources**

| **SNP** | **Beta** | **Standard error** | **P-value** | **Effect allele frequency** | **Effect allele** | **Other allele** | **Outcome \|\| GWAS Pubmed or OpenGWAS id** |
| --- | --- | --- | --- | --- | --- | --- | --- |
| rs2606345 | 0.003 | 0.009 | 0.751 | 0.538 | A | C | Schizophrenia \|\| Open GWAS id:ieu-b-5099 |
| rs10275488 | -0.002 | 0.011 | 0.825 | 0.139 | T | C | Schizophrenia \|\| Open GWAS id:ieu-b-5099 |
| rs10275488 | 0.005 | 0.007 | 0.450 | 0.118 | T | C | Major depression \|\| Open GWAS id:ieu-b-102 |
| rs2472297 | 0.015 | 0.010 | 0.125 | 0.220 | T | C | Schizophrenia \|\| Open GWAS id:ieu-b-5099 |
| rs12903896 | -0.018 | 0.008 | 0.024 | 0.522 | T | C | Schizophrenia \|\| Open GWAS id:ieu-b-5099 |
| rs2606345 | 0.011 | 0.005 | 0.017 | 0.675 | A | C | Major depression \|\| Open GWAS id:ieu-b-102 |
| rs4410790 | -0.003 | 0.008 | 0.697 | 0.578 | C | T | Schizophrenia \|\| Open GWAS id:ieu-b-5099 |
| rs10950657 | -0.005 | 0.008 | 0.493 | 0.496 | A | C | Schizophrenia \|\| Open GWAS id:ieu-b-5099 |
| rs35686934 | 0.012 | 0.010 | 0.245 | 0.057 | A | G | Major depression \|\| Open GWAS id:ieu-b-102 |
| rs12903896 | -0.002 | 0.005 | 0.681 | 0.654 | T | C | Major depression \|\| Open GWAS id:ieu-b-102 |
| rs4410790 | 0.005 | 0.005 | 0.241 | 0.631 | C | T | Major depression \|\| Open GWAS id:ieu-b-102 |
| rs73083829 | -0.001 | 0.006 | 0.899 | 0.168 | A | C | Major depression \|\| Open GWAS id:ieu-b-102 |
| rs73083829 | -0.005 | 0.010 | 0.612 | 0.183 | A | C | Schizophrenia \|\| Open GWAS id:ieu-b-5099 |
| rs2472297 | 0.010 | 0.005 | 0.043 | 0.263 | T | C | Major depression \|\| Open GWAS id:ieu-b-102 |
| rs10950657 | -0.002 | 0.004 | 0.631 | 0.530 | A | C | Major depression \|\| Open GWAS id:ieu-b-102 |
| rs2472297 | 0.019 | 0.016 | 0.233 | NA | C | T | Anorexia nervosa\|\| Pubmed id: 31308545 |
| rs12903896 | 0.036 | 0.014 | 0.010 | NA | C | T | Anorexia nervosa\|\| Pubmed id: 31308545 |
| rs2606345 | 0.002 | 0.015 | 0.905 | NA | C | A | Anorexia nervosa\|\| Pubmed id: 31308545 |
| rs4410790 | 0.008 | 0.014 | 0.573 | NA | T | C | Anorexia nervosa\|\| Pubmed id: 31308545 |
| rs10950657 | -0.002 | 0.014 | 0.864 | NA | C | A | Anorexia nervosa\|\| Pubmed id: 31308545 |
| rs73083829 | -0.012 | 0.019 | 0.519 | NA | C | A | Anorexia nervosa\|\| Pubmed id: 31308545 |
| rs10275488 | -0.007 | 0.022 | 0.750 | NA | C | T | Anorexia nervosa\|\| Pubmed id: 31308545 |
| rs4410790 | -0.007 | 0.010 | 0.466 | NA | T | C | Bipolar disorder \|\| Pubmed id: 34002096 |
| rs10950657 | -0.009 | 0.009 | 0.337 | NA | C | A | Bipolar disorder \|\| Pubmed id: 34002096 |
| rs73083829 | 0.012 | 0.013 | 0.370 | NA | C | A | Bipolar disorder \|\| Pubmed id: 34002096 |
| rs10275488 | 0.019 | 0.015 | 0.221 | NA | C | T | Bipolar disorder \|\| Pubmed id: 34002096 |
| rs2606345 | -0.032 | 0.010 | 0.002 | NA | C | A | Bipolar disorder \|\| Pubmed id: 34002096 |
| rs2472297 | -0.024 | 0.011 | 0.026 | NA | C | T | Bipolar disorder \|\| Pubmed id: 34002096 |
| rs12903896 | 0.019 | 0.010 | 0.054 | NA | C | T | Bipolar disorder \|\| Pubmed id: 34002096 |

## **Table S4: Meta-analyzed variant-outcome associations and Mendelian randomization Wald ratios**

| Outcome | SNP | Gene | Effect allele | Other allele | Meta-analysed outcome beta | Outcome standard error | Outcome p-value | Wald ratio | Wald ratio standard error |
| --- | --- | --- | --- | --- | --- | --- | --- | --- | --- |
| Schizophrenia | rs10275488 | AHR | T | C | -0.006 | 0.011 | 0.585 | -0.046 | 0.085 |
| Schizophrenia | rs10950657 | AHR | A | C | -0.005 | 0.007 | 0.533 | 0.067 | -0.108 |
| Schizophrenia | rs12903896 | CYP1A2 | T | C | -0.016 | 0.008 | 0.045 | 0.206 | -0.103 |
| Schizophrenia | rs2472297 | CYP1A2 | T | C | 0.018 | 0.010 | 0.061 | -0.114 | -0.061 |
| Schizophrenia | rs2606345 | CYP1A2 | A | C | 0.005 | 0.008 | 0.543 | -0.071 | -0.117 |
| Schizophrenia | rs4410790 | AHR | T | C | 0.001 | 0.007 | 0.892 | 0.010 | 0.070 |
| Schizophrenia | rs73083829 | AHR | A | C | -0.005 | 0.009 | 0.596 | -0.057 | 0.108 |
| Bipolar disorder | rs10275488 | AHR | T | C | -0.014 | 0.014 | 0.295 | -0.115 | 0.110 |
| Bipolar disorder | rs10950657 | AHR | A | C | 0.010 | 0.009 | 0.239 | -0.149 | -0.126 |
| Bipolar disorder | rs12903896 | CYP1A2 | T | C | -0.016 | 0.009 | 0.067 | 0.211 | -0.115 |
| Bipolar disorder | rs2472297 | CYP1A2 | T | C | 0.020 | 0.010 | 0.043 | -0.127 | -0.063 |
| Bipolar disorder | rs2606345 | CYP1A2 | A | C | 0.022 | 0.009 | 0.014 | -0.312 | -0.127 |
| Bipolar disorder | rs4410790 | AHR | T | C | -0.007 | 0.009 | 0.402 | -0.071 | 0.085 |
| Bipolar disorder | rs73083829 | AHR | A | C | -0.008 | 0.012 | 0.472 | -0.097 | 0.135 |
| Major depression | rs10275488 | AHR | T | C | 0.004 | 0.006 | 0.487 | 0.033 | 0.047 |
| Major depression | rs10950657 | AHR | A | C | -0.001 | 0.004 | 0.711 | 0.021 | -0.057 |
| Major depression | rs12903896 | CYP1A2 | T | C | -0.001 | 0.004 | 0.718 | 0.019 | -0.051 |
| Major depression | rs2472297 | CYP1A2 | T | C | 0.009 | 0.004 | 0.033 | -0.059 | -0.028 |
| Major depression | rs2606345 | CYP1A2 | A | C | 0.008 | 0.004 | 0.042 | -0.113 | -0.056 |
| Major depression | rs35686934 | CYP1A2 | A | G | 0.004 | 0.008 | 0.595 | -0.027 | -0.051 |
| Major depression | rs4410790 | AHR | T | C | -0.004 | 0.004 | 0.345 | -0.036 | 0.038 |
| Major depression | rs73083829 | AHR | A | C | -0.002 | 0.005 | 0.749 | -0.019 | 0.059 |
| Anorexia nervosa | rs10275488 | AHR | T | C | 0.011 | 0.022 | 0.611 | 0.088 | 0.174 |
| Anorexia nervosa | rs10950657 | AHR | A | C | -0.001 | 0.014 | 0.917 | 0.021 | -0.204 |
| Anorexia nervosa | rs12903896 | CYP1A2 | T | C | -0.036 | 0.014 | 0.010 | 0.467 | -0.181 |
| Anorexia nervosa | rs2472297 | CYP1A2 | T | C | -0.017 | 0.016 | 0.275 | 0.108 | -0.099 |
| Anorexia nervosa | rs2606345 | CYP1A2 | A | C | 0.002 | 0.015 | 0.896 | -0.027 | -0.210 |
| Anorexia nervosa | rs4410790 | AHR | T | C | 0.009 | 0.014 | 0.519 | 0.084 | 0.131 |
| Anorexia nervosa | rs73083829 | AHR | A | C | 0.014 | 0.019 | 0.445 | 0.165 | 0.215 |

# **Supplementary Figures**

## **Fig. S1. X-Y scatter graphs of Mendelian randomization results**

| 1. Anorexia | 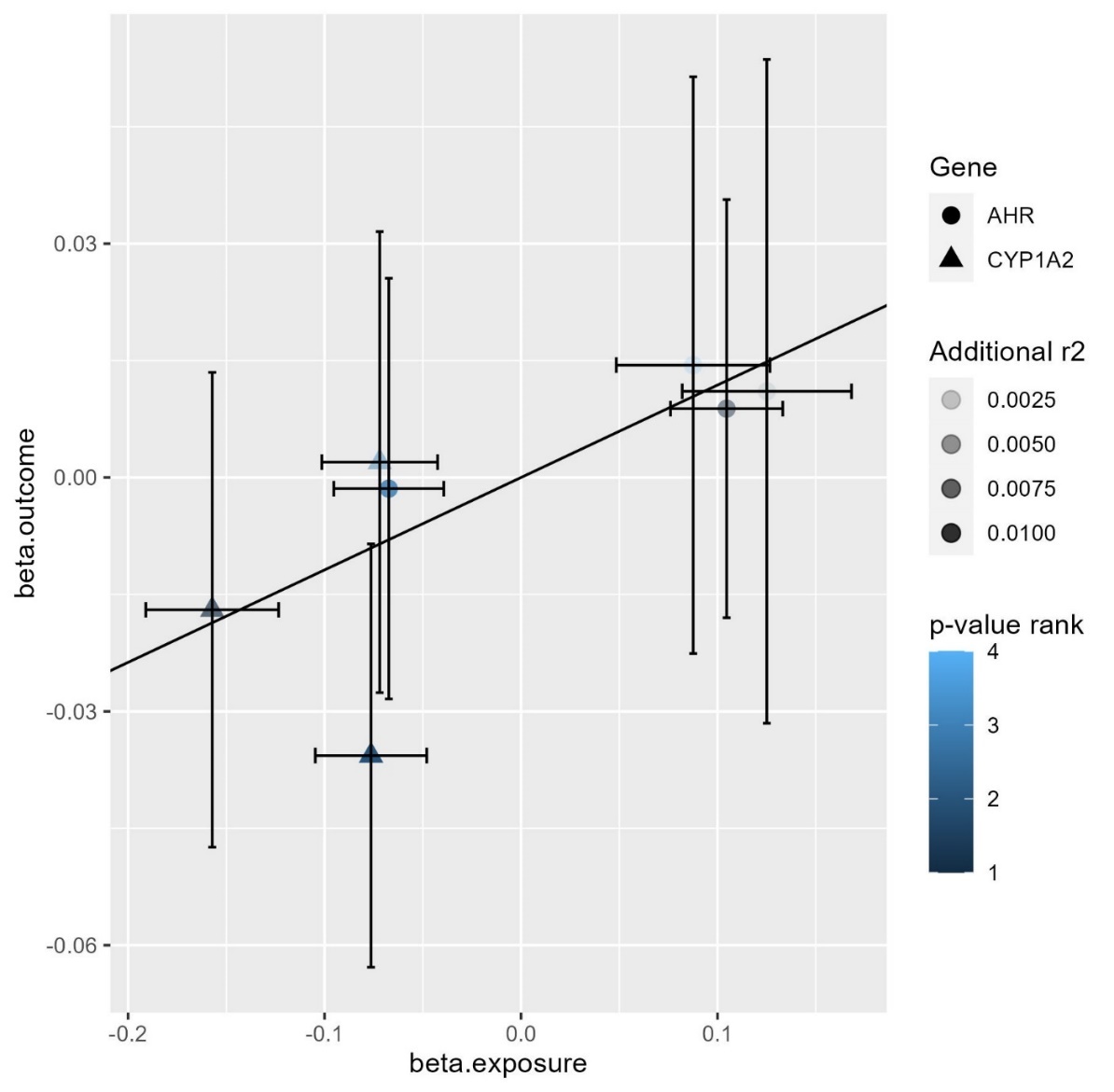 |
| --- | --- |
| B) Bipolar | 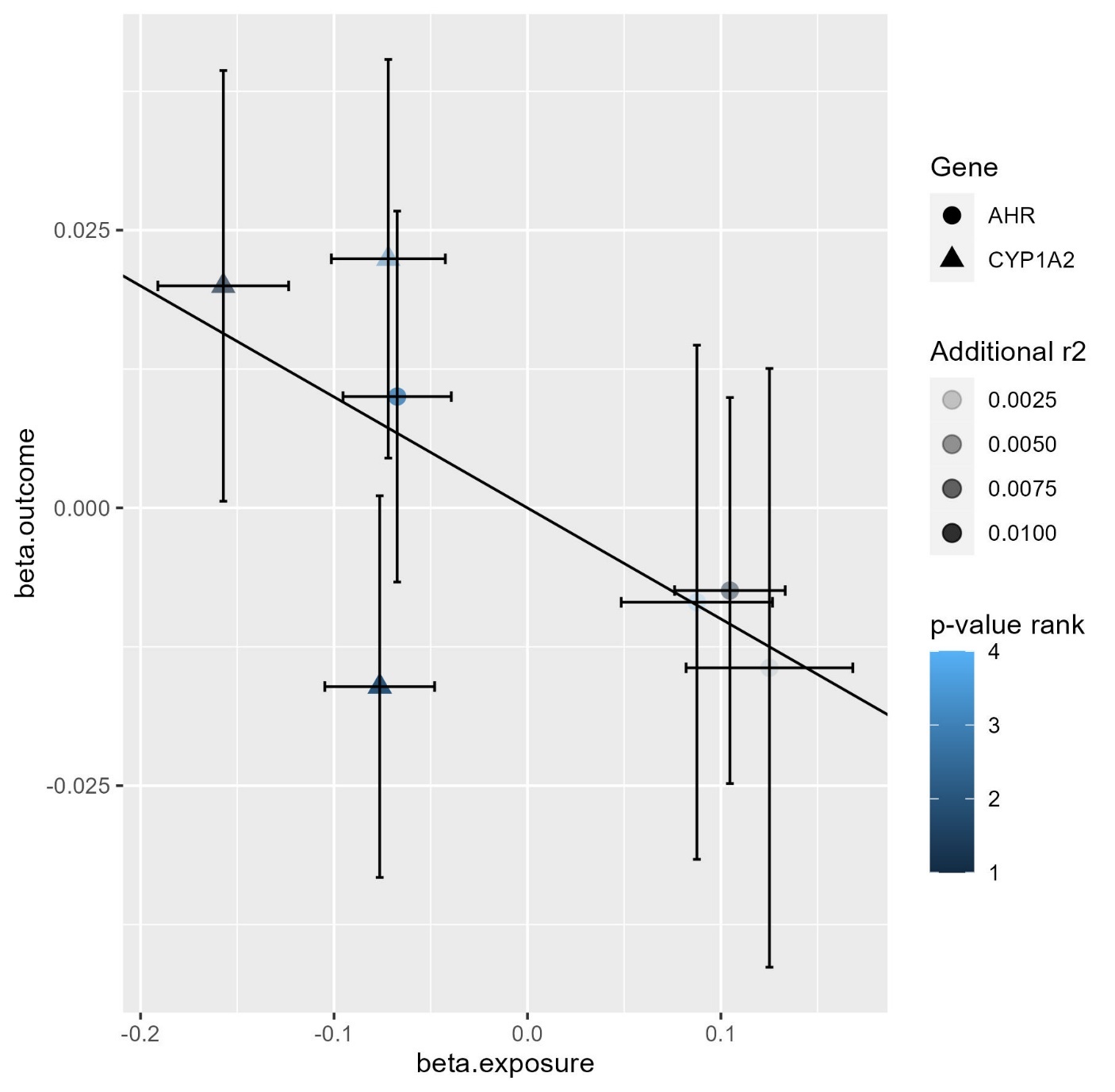 |
| C) MDD | 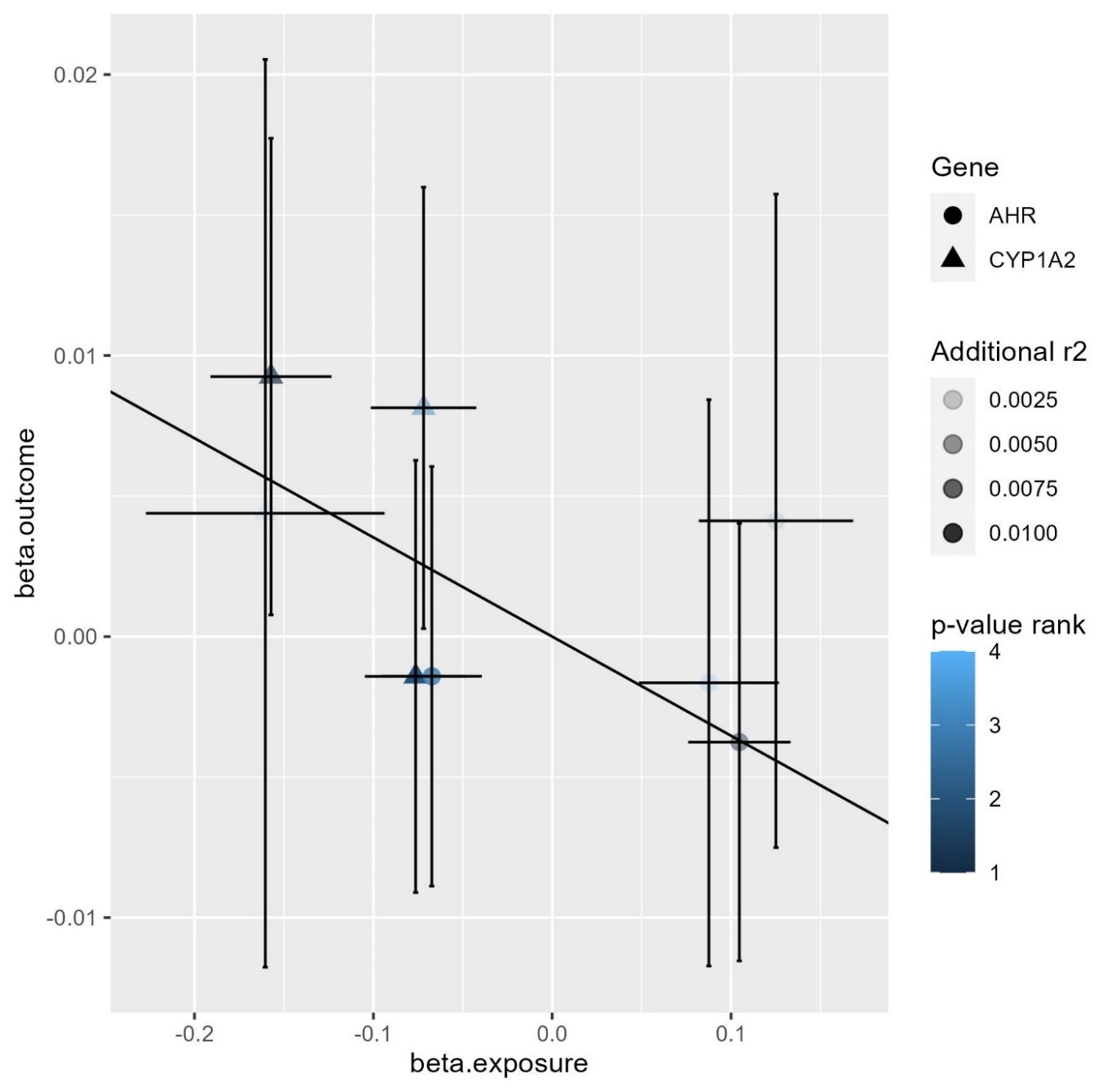 |
| D) Schizophrenia | 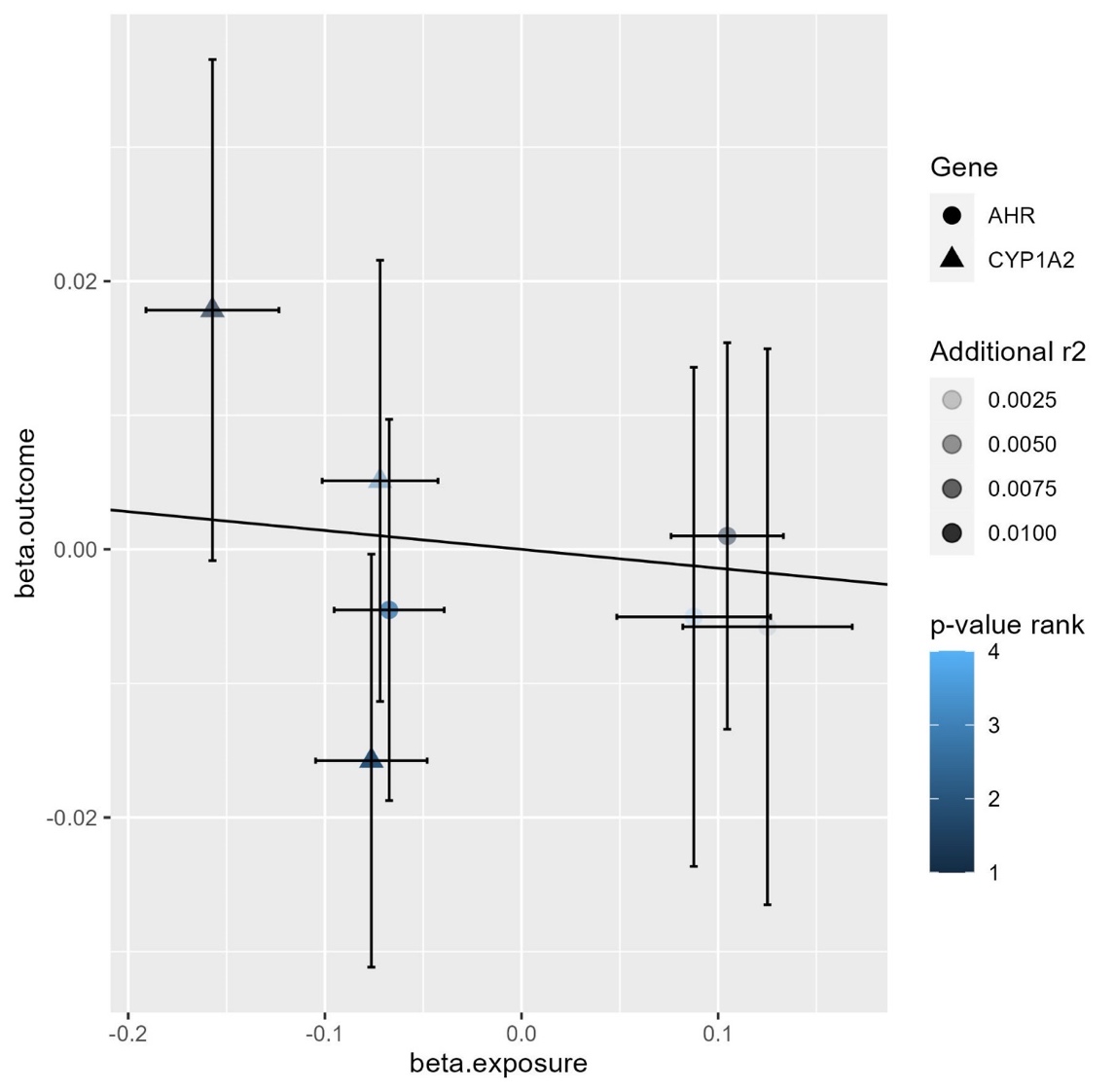 |

Additional r2 = the variance explained by the variant in addition to the variance explained by variants with smaller p-values. p-value rank = the rank of the SNPs within each gene region so that the SNP with the smallest variant-exposure p-value has a rank of 1, the second smallest a rank of 2, and so on. A naive inspection of SNP clustering may be misleading could be misleading because the observations are not independent of each other. Instead, less weight should be given to SNPs which contribute less to the analysis (i.e., high ranks and small additional variance explained). MDD: major depressive disorder; SNP: single nucleotide polymorphism.

## **Fig. S2. Leave-one-out analyses**

| 1. Anorexia nervosa | Gene-specific  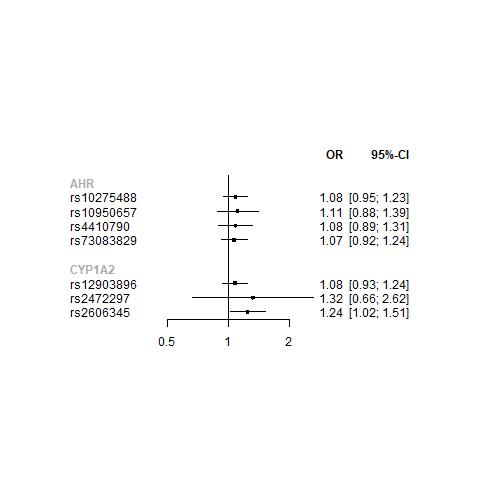  Combined  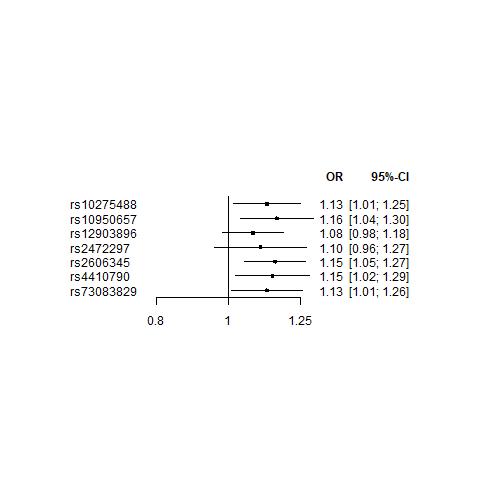 |
| --- | --- |
| 1. Bipolar | Gene-specific  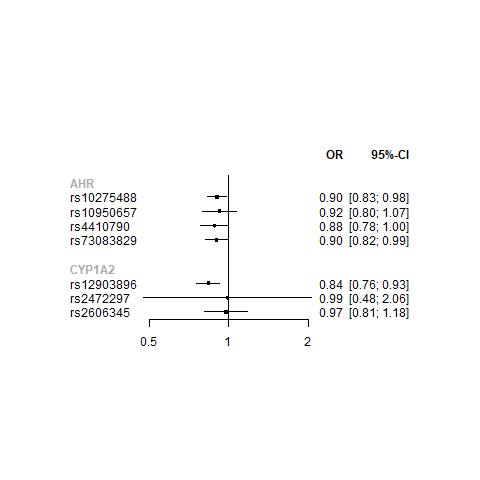  Combined  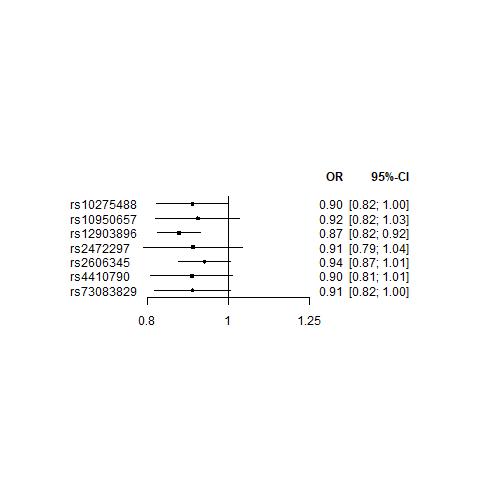 |
| 1. Major Depressive disorder | Gene-specific  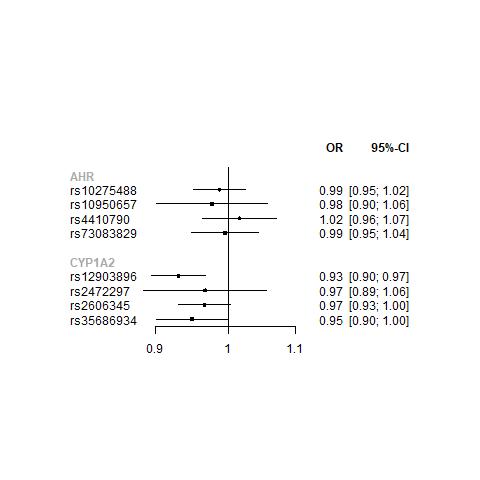  Combined  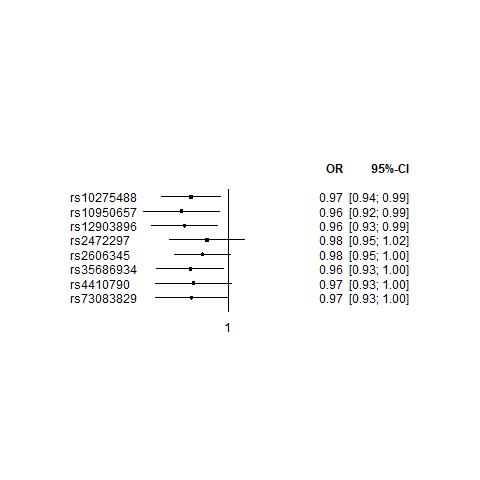 |
| 1. Schizophrenia | Gene-specific  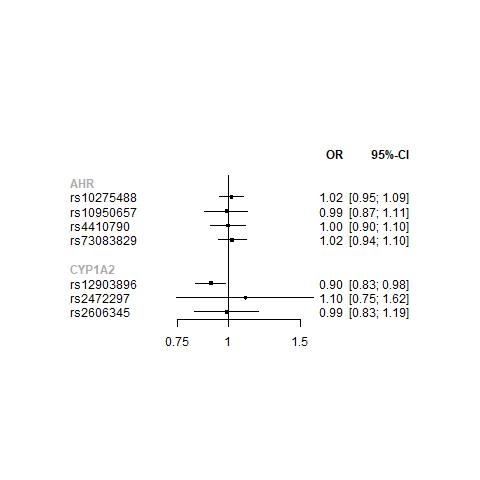 Combined  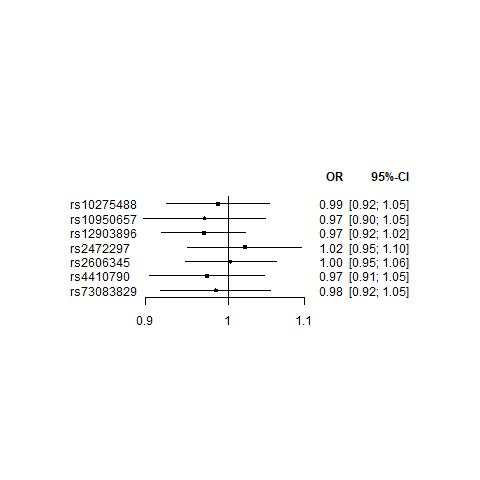 |
